# Identification of SCN7A as the key gene associated with tumor mutation burden in gastric cancer

**DOI:** 10.1186/s12876-022-02112-4

**Published:** 2022-02-05

**Authors:** Wenjie Li, Kezhi Zhou, Mengting Li, Qian Hu, Wanhui Wei, Lan Liu, Qiu Zhao

**Affiliations:** 1 Department of Gastroenterology, Zhongnan Hospital of Wuhan University, Wuhan, China; 2 Hubei Clinical Center and Key Lab of Intestinal and Colorectal Diseases, Wuhan, China

**Keywords:** Gastric cancer, TMB, SCN7A

## Abstract

**Objective:**

Previous studies have shown that tumor mutation burden (TMB) in cancer is associated with prognosis. The purpose of this study is to identify TMB related genes in gastric cancer (GC) and to explore their prognostic value.

**Methods:**

In our research, weighted gene coexpression network analysis (WGCNA) algorithm was used to cluster the most relevant TMB modules in the Cancer Genome Atlas (TCGA) database. Limma package was used to screen the differentially expressed genes, and the intersection was identified as hub genes. We used gene expression profiling interactive analysis (GEPIA) and survival algorithm to analyze the clinical characteristics and prognosis of hub genes in tumor and normal tissue samples of TCGA and Gene Expression Omnibus cohort respectively. We also used CIBERSORT algorithm to calculate the proportion of 22 tumor immune cells in the high and low expression subgroups of hub genes. In addition, we used gene set enrichment analysis (GSEA) to predict the biological function of hub genes. *P* < 0.05 was considered statistically significant.

**Results:**

In the TCGA cohort, TMB was significantly correlated with the clinical features of GC (*P* < 0.05). Through WGCNA and differential gene analysis, we identified SCN7A as the hub gene (*P* < 0.05, |log2fc|> 1, and mm > 0.8). We found that the expression of SCN7A in tumor tissues was lower than that in normal tissues, and its expression level was also related to overall survival rate and tumor stage. GSEA analysis showed that SCN7A low expression group was enriched with "DNA replication", "base extension repair" and "proteasome" gene sets in GC. In addition, we found that there were significant differences in the infiltration degree of 7 kinds of immune cells between the two groups.

**Conclusion:**

TMB can indicate the prognosis of gastric cancer. SCN7A is a hub gene associated with TMB, and its low expression is associated with better prognosis.

**Supplementary Information:**

The online version contains supplementary material available at 10.1186/s12876-022-02112-4.

## Introduction

Gastric cancer is a malignant disease that occurred over 1 million and led to about 783,000 deaths worldwide in 2018, making it the fifth most common cancer and the third leading cause of tumor-related death [1]. With the improvement of diet and the screening and elimination of Helicobacter pylori, the incidence of gastric cancer has decreased. However, the GC incidence decreased among older adults and increased among younger individuals, especially in female adults [2]. The proportion of proximal gastric cancer, which usually leads to poor prognosis, has also increased. The current therapy for GC is primarily surgery resection combined with or without chemotherapy, which is limited. Hence, it is vital to find new strategies to improve prognosis. Although GC is a disease with high heterogeneity[3], a high incidence of somatic mutation occurred in GC patients [4]. Immunotherapy blocking immune checkpoints have achieved considerable progress in malignant diseases with increased somatic mutations, such as Hodgkin's lymphoma, melanoma and non-small cell lung cancer [5–7]. Thus, immunotherapy is a promising therapy for GC.

Tumor mutation burden (TMB) is defined as the number of mutations, including replacement and insertion/deletion, existing within a megabase of genomic territory [8]. It has been suggested by several studies that TMB is associated with the efficacy of immunotherapy, and patients with high TMB can benefit from immunotherapy and gain prolonged survival time in multiple cancer types [9–13]. However, immunotherapy does not suit every patient. At present, several clinical trials indicated that immunotherapy did not perform satisfactorily when treating GC patients [14, 15]. Therefore, finding novel biomarker for screening patients who are able to benefit from immunotherapy is of great importance.

The weighted gene coexpression network analysis (WGCNA) is a R package comprehensively collecting R functions for performing weighted correlation network analysis [16]. With the development of high-throughput sequencing technology, WGCNA is widely used to analyze data and microarray data to identity key genes in various diseases. For example, Nangraj, AS et al. applied the WGCNA to identity five hub genes between Barrett's esophagus and Esophageal adenocarcinoma [17].

In this study, TMB was calculated in 433 patients with GC in the TCGA database. Then, the WGCNA was utilized to identify hub gene associated with TMB and overall survival of GC patients. The Kyoto Encyclopedia of Genes and Genomes (KEGG) pathway enrichment analysis and Gene Ontology (GO) analysis were used to explore potential mechanisms. With the validation of external dataset GSE62254, we finally dig out the biomarker related to TMB in GC patients.

## Materials and methods

### Data preparation

The human GC mRNA expression data and relevant clinical data were downloaded from the TCGA database (https://tcga-data.nci.nih. gov/tcga/). Somatic mutation data were also downloaded from the TCGA database. The GSE62254 GC mRNA expression data and corresponding clinical data were downloaded from (Gene Expression Omnibus) GEO (https://www.ncbi.nlm.nih.gov/geo/).

### Calculation of TMB in GC patients

TMB is defined as the total number of mutations including somatic, coding, base replacement, and insert-deletion mutations per megabase in tumor tissue. 'maftools’ R package was applied to calculate the TMB of each sample in TCGA-STAD, and the samples were divided into low and high TMB subgroups based on the median value. Moreover, we analyzed the difference between TMB high and low group among variant clinical characteristics.

### Co-expression network construction

We calculated variances of 19,525 genes from “TCGA-STAD.” Ranked by decreasing standard deviation (SD), the top 25% of genes (n = 4881) were selected for further analysis. The “WGCNA” R package was used to construct a co-expression network of the above genes [18]. Further, the pickSoftThreshold function was used to estimate the soft-thresholding power β for constructing modules. The parameter *β* is a soft-threshold that can stress strong correlations between genes while penalizing weak correlations. According to the scale-free topology fit index and the mean connectivity (Additional file 1: Fig. S2 A–D), the value closest to 0.8 was chosen as the power to establish a scale-free network (β = 3, scale R^2^ = 0.8). Next, to measure the network connectivity of genes, the adjacency was converted into a topological overlap matrix (TOM) [19]. Genes with similar expression patterns were classified into diverse modules with the smallest gene size of 30 based on the topological overlap matrix similarity. Eventually, the correlation between the module eigengenes and the clinic traits (overall survival, survival status, or TMB) was estimated to identify the correlative modules. Gene significance(GS) was defined as the absolute value of the correlation between genes and clinical traits. Quantitative measurement of the module relationship was determined as the connection strength between eigengenes in each module and gene expression profiles. The module significance (MS) was defined as the average GS for all the genes in the significant module. Moreover, module eigengene (ME) was the major component in the principal component analysis for each module, which could categorize all gene expression patterns into a single characteristic expression profile within a specific module. Based on the highest MS and the closest MEs correlation with TMB, the key module was identified.

### Differentially expressed genes screening

The ‘limma’ R package was used to identify differentially expressed gene (DEGs) between normal and tumor samples [20]. Under the threshold of *p*-value < 0.05 and |log2FC| > 1, 304 significant DEGs were selected for further analysis.

### Functional enrichment analysis and hub gene identification

To further investigate the function of DEGs in the key module, common genes in the key module and DEGs were exported. We used ‘‘clusterProfiler’’ R package to perform GO and KEGG enrichment analysis of the genes [21]. The enrichments of GO and KEGG analysis with *p* < 0.05 were presented by using ‘‘ggplot2’’ R package. Moreover, the genes in the key module were ranked by decreasing module membership (MM) value and genes with MM > 0.8 were identified as hub genes.hub genes were identified with high module membership (MM) which means genes were significantly related with the traits.

### Hub gene validation

The survival curve was based on Gene expression profiling interactive analysis (GEPIA, http://gepia.cancer-pku.cn/) with TCGA data. The overall survival analysis of GSE62254 was performed by “survival” R package. Expression of the hub gene between tumor and normal samples and among clinical traits were analyzed and visualized by GraphPad Prism software. The relationship between expression and clinical traits was evaluated by Chi-square analysis by using SPSS 25.0. The statistical significance was evaluated by two-tailed Student’s t-tests. *p* < 0.05 was considered statistical significance.

### Gene set enrichment analysis

Three hundred and seventy-four samples were divided into high and low groups according to the expression of SCN7A. Gene set enrichment analysis was applied to enrich the KEGG pathways related to the two groups. | Normalized enrichment score |> 1.8, p-value < 0.05, and gene size > 30 were used as the cutoff criteria.

### Evaluation of tumor-infiltrating immune cells

The CIBERSORT algorithm was utilized to estimate the composition of tumor-infiltrating immune cells in normal tissues and GC tissues from the TCGA database [22]. The CIBERSORT algorithm containing 547 marker genes' expression signature matrix can be used to calculate 22 immune cells regarding LM22. LM22 is a txt defining 22 subtypes of immune cells referring to the annotated gene signature matrix, downloaded from the CIBERSORT website portal (Http:// cibersort.stanford.edu/).

## Results

### Tumor mutation burden spectrum and its correlation with clinical characteristics

**Fig. 1:**
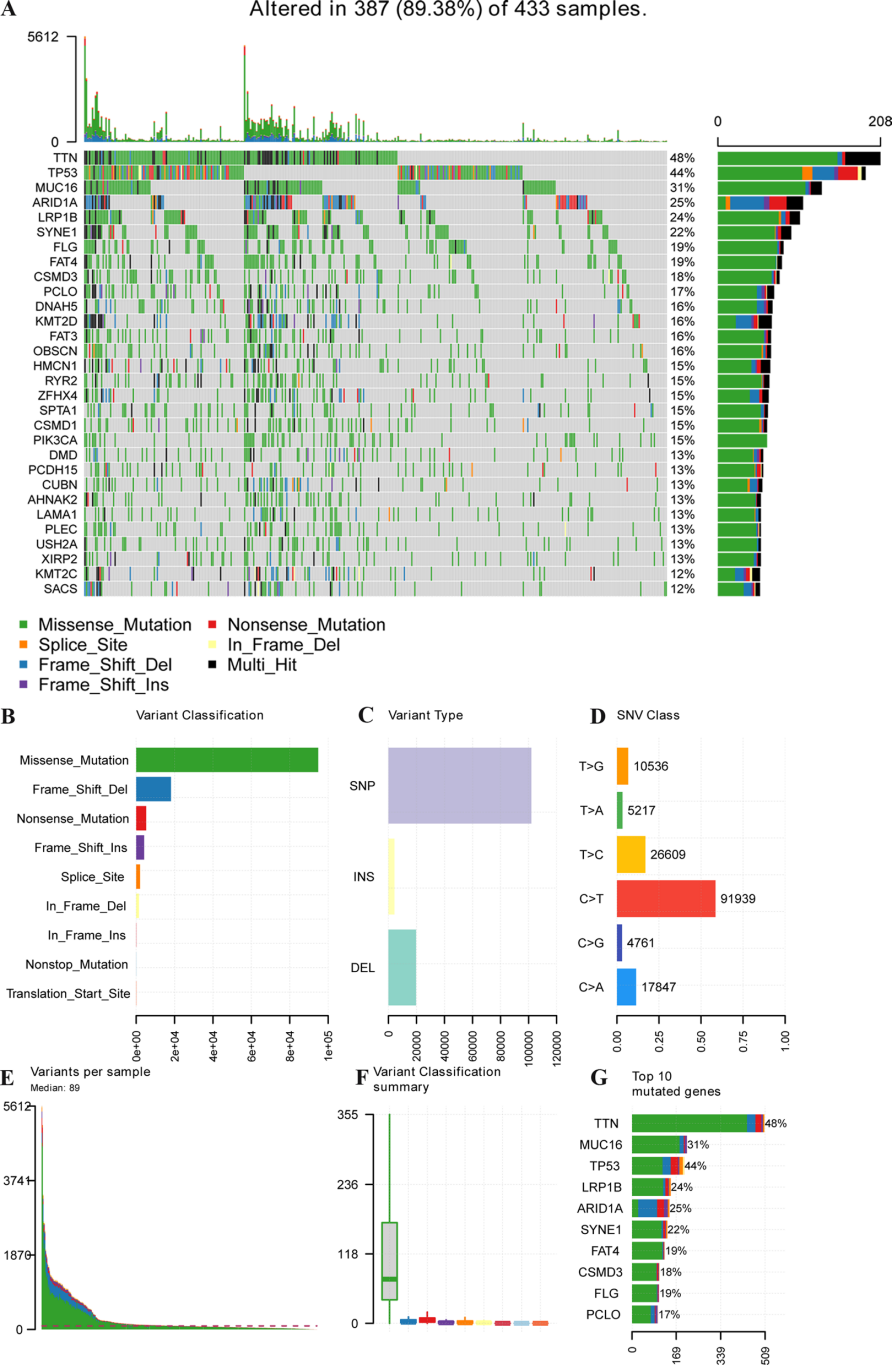
TCGA GC mutation cohort. **A** Oncoplot depicts the frequently mutated genes in gastric cancer from TCGA cohort. The left panel shows mutation frequency, and genes are ordered by their mutation frequencies. The bottom panel presents different mutation types. **B**–**G** Landscape of TCGA GC cohort mutations

We found that among 33 types of tumors, the TMB level of GC was relatively high (Additional file 2: Fig. S1A). The waterfall chart showed the top 30 mutated genes and related mutation types in TCGA-STAD (Fig. 1A). Missense mutations were the main type of mutation that occurred in GC patients (Fig. 1B). Single nucleotide polymorphisms occurred more than insertions or deletions (Fig. 1C, D), and C > T was the common single-nucleotide variation (SNV) in GC. The frequencies of mutation in each sample were shown in Fig. 1E and F. The barplot reveals the mutation of top 10 mutated genes (Fig. 1G).

**Fig. 2:**
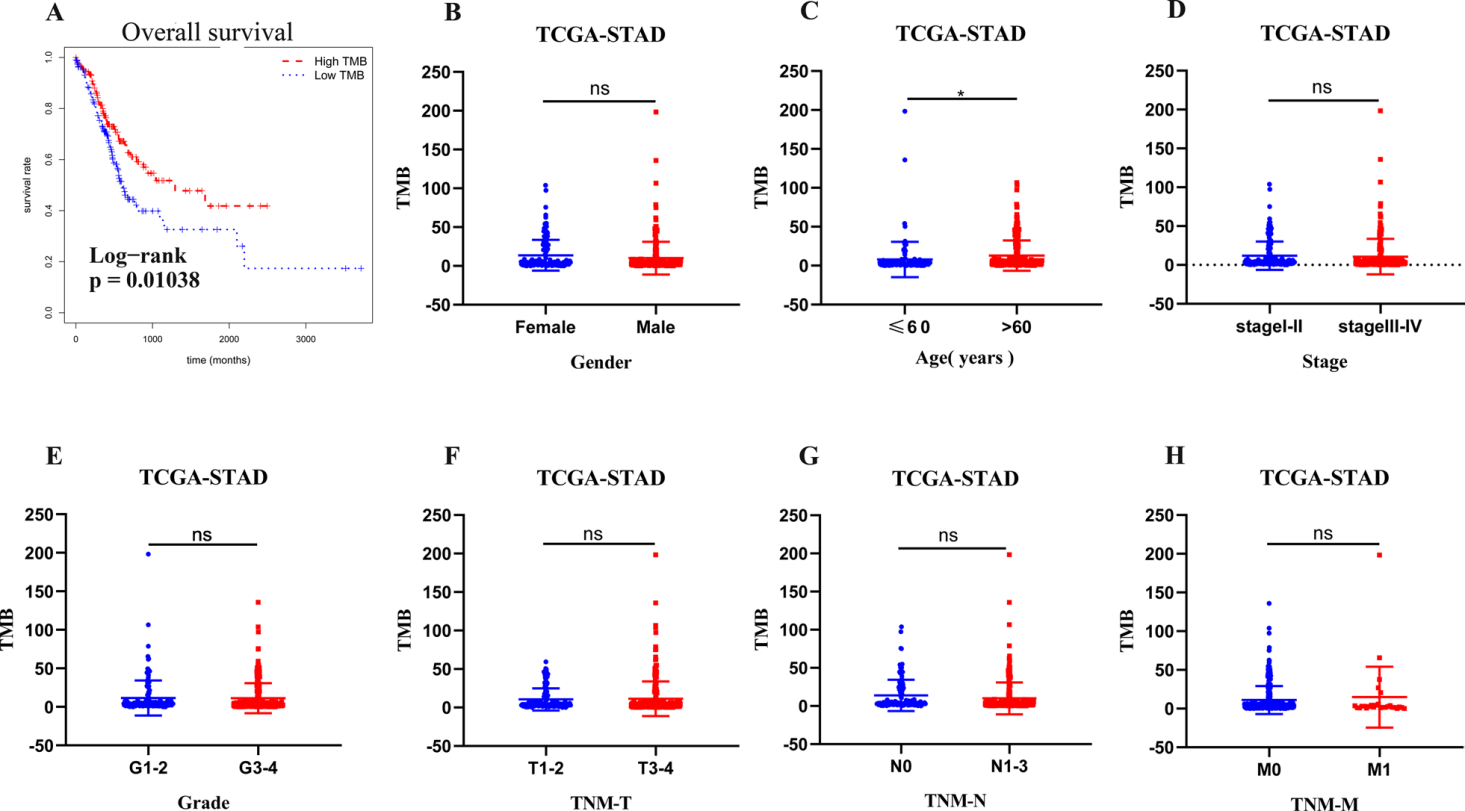
Clinical significance of tumor mutation burden in gastric cancer patients. **A** Survival analysis to explore the overall survival of gastric cancer patients between the high and low tumor mutation burden groups. **B**–**H** Correlation between tumor mutation burden values and clinical characteristics in gastric cancer

To confirm the significance of TMB, we analyzed the relationship between TMB and clinical characteristics. The survival plot (Fig. 2A) showed that high-TMB was associated with better clinical outcome. TMB did not differ significantly among different gender, stage, grade, and Tumor Node Metastasis (TNM) (Fig. 2B, D–H). The level of TMB in patients > 60 years old was remarkably higher than patients ≤ 60 (Fig. 2C).

### Co-expression network construction and key module identification

**Fig. 3:**
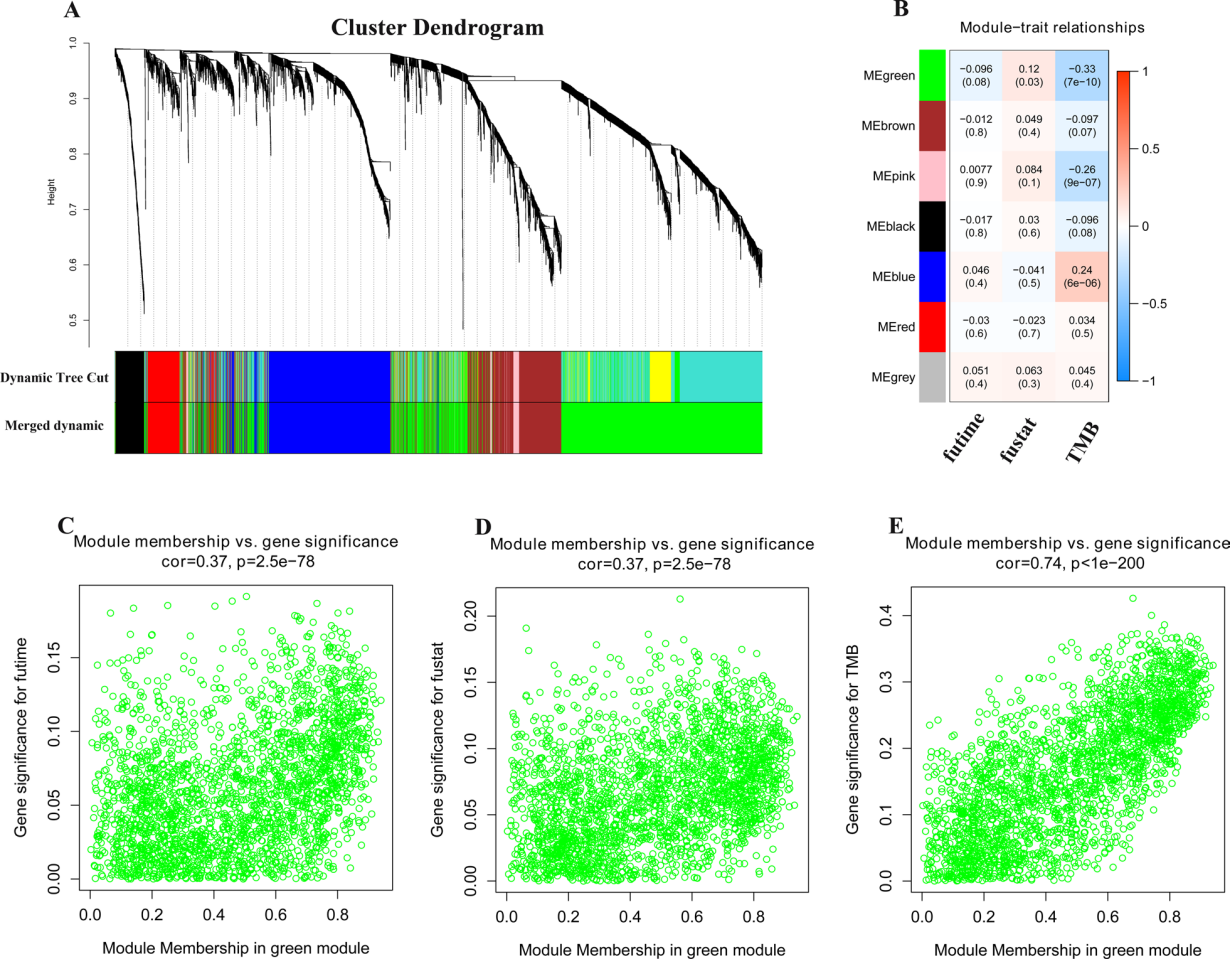
Establishing correlation patterns among differentially expressed genes (n = 4881) in gastric cancer using Weighted Gene Co-expression Network (WGCNA) analysis. **A** Hierarchical clustering of genes with dissimilarity based on topological overlap are shown along with the modules detected and the merged modules. **B** Heatmap for the correlation between modules and clinical traits. **C**–**E** The scatter plot of module eigengenes in the green module

We calculated the variances of 19,525 genes and chose 4881 genes with variances more than quartiles of variances for the subsequent analysis. R package ‘‘WGCNA’’ was applied to compute the 4881 genes into modules through the average linkage hierarchical clustering, and seven modules were finally presented with different colors (Fig. 3A). Among the modules, the maximum module contains 2387 genes (green). Moreover, the green module exhibits highest correlation with TMB (r = − 0.33, *p* = 7e−10) and significantly correlated with patients’ survival status (r = 0.12, *p* = 0.03) (Fig. 3B). Figure 3C–E showed the correlation between module membership and gene significance in the green module. The results indicated that the membership in the green module was significantly correlated with overall survival (r = 0.37), patients’ status (r = 0.37) and TMB (r = 0.74). Thus, genes in the green module were extracted for hub gene hunting.

### Hub gene identification and validation

**Fig. 4:**
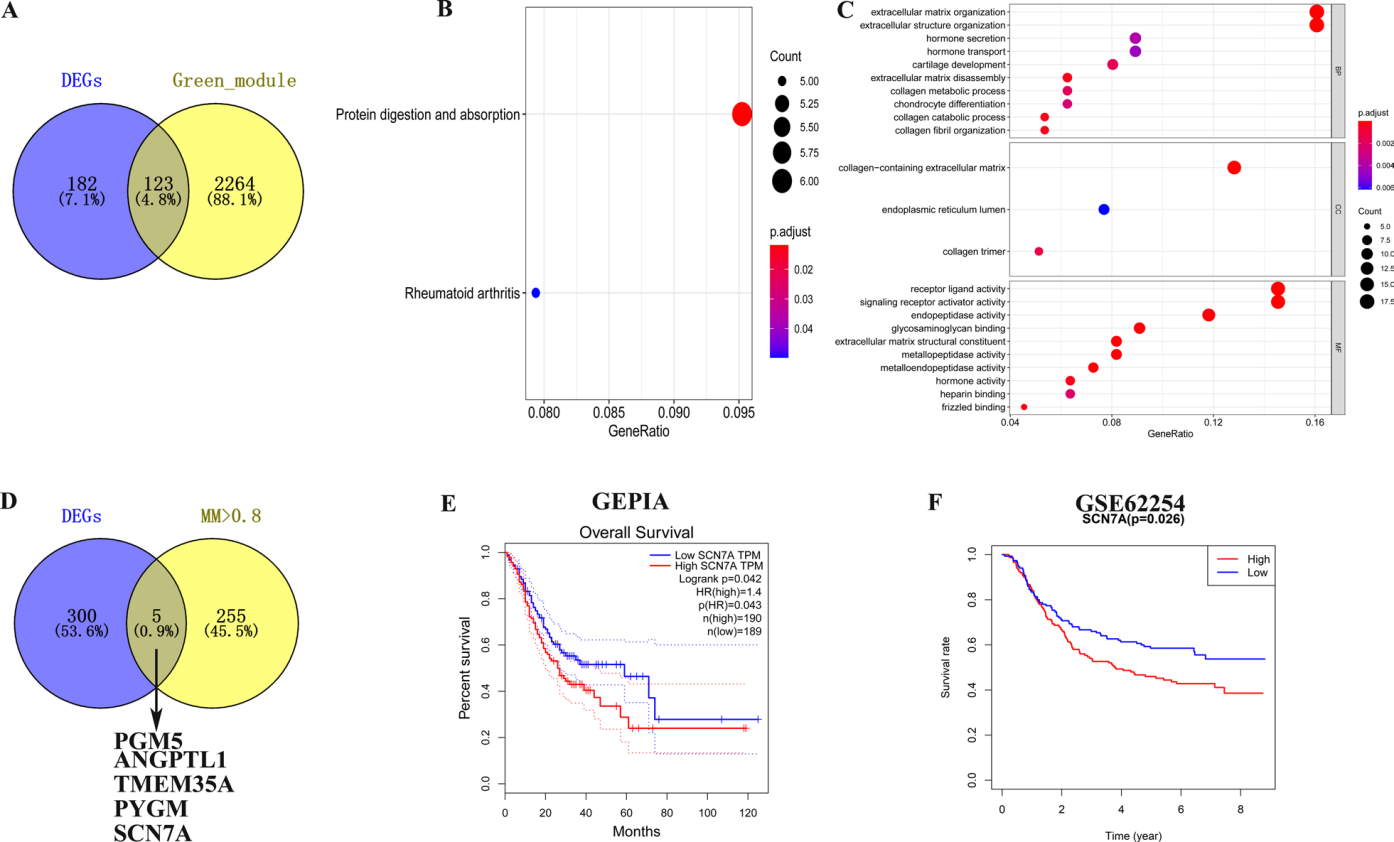
**A** Overlap of green module hub genes and DEGs genes. **B** KEGG pathway enrichment analysis. **C** GO pathway enrichment analysis. **D** Overlap of DEGs genes and green module genes (MM > 0.8). **E**, **F** The overall survival of patients with high and low expression levels of SCN7A by GEPIA and in GSE62254

To further identify the hub gene, the threshold of *p*-value < 0.05 and |log2FC| > 1 were selected to identify DEGs. 153 upregulated genes and 151 downregulated genes were chosen for further analysis. 123 DEGs were also in the green module (Fig. 4A). To further evaluate the function of 123 DEGs, GO and KEGG pathway analyses were performed. Protein digestion and absorption pathway was the most remarkably enriched KEGG pathway (Fig. 4B). “Extracellular matric organization” and “extracellular structure organization” were the most enriched in biological process (BP) while “collagen-containing extracellular matrix” was the most significantly enriched in cellular component (CC) and “receptor ligand activity” and “signalling receptor activator activity” in the molecular function (MF) (Fig. 4C).

**Fig. 5:**
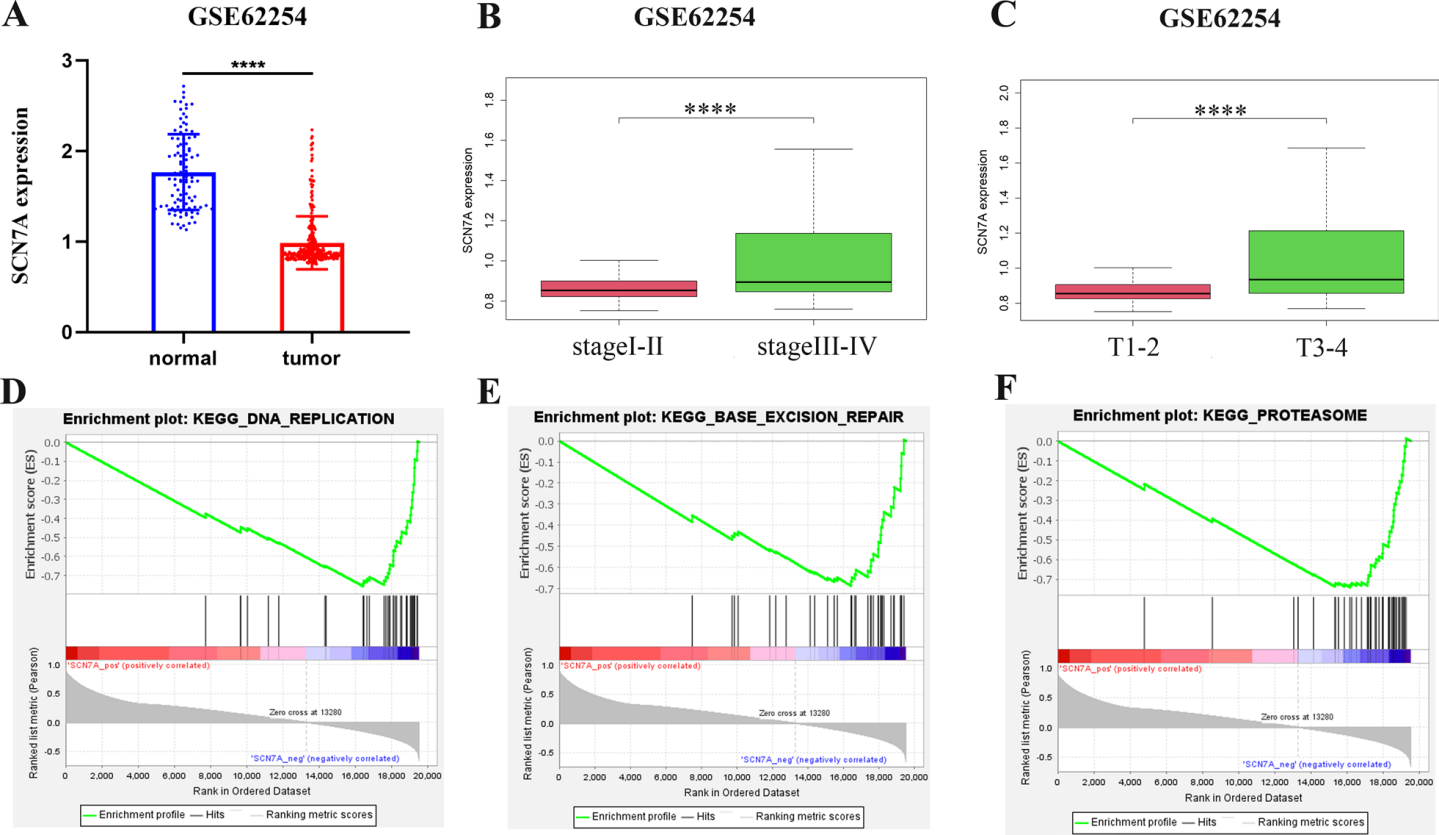
**A** Expression levels of SCN7A in normal group and GC groups in GSE62254. **B**, **C** Expression levels of SCN7A in different clinical and pathological stage in GSE62254. Gene enrichment plots shows that a series of gene sets including **D** DNA replication, **E** base excision repair, and **F** proteasome

Furthermore, to identify genes that were highly connected within a module, we set MM > 0.8 as the threshold, and 5 genes were finally selected as the candidates (Fig. 4D). The survival plot by GEPIA revealed low expression level of SCN7A was associated with overall survival rate (Fig. 4E, *p* = 0.043) and further validated by external dataset GSE62254 (Fig. 4F, *p* = 0.026). Thus, SCN7A was identified as the hub gene. Furthermore, we explored the relationship between SCN7A and clinical characteristics in GSE62254. The expression level of SCN7A in tumor tissues was lower than normal tissues (Fig. 5A). We also found that the expression of SCN7A was lower in stage I-II compared with stage III-IV (Fig. 5B) while it is higher in patients with T3-4 (Fig. 5C). In addition, the samples in GSE62254 were divided into two groups (high and low) by SCN7A average expression level. The relationship between clinical traits and the expression level of SCN7A was analyzed. As shown in Table 1, the expression level of SCN7A was related with gender, age, TNM-T, TNM-N and stage (with *p* < 0.01). To investigate the potential biological function of SCN7A in GC samples, we performed GSEA and with the threshold of |NES| > 1.8 and NP < 0.05, the gene set of “DNA REPLICATION”, “BASE EXTENSION REPAIR” and “PROTEASOME” was enriched in SCN7A lowly expressed samples (Fig. 5D–F). In summary, our results indicated that SCN7A was associated with clinical characteristics and TMB to a large extent.

**Table 1:** Characteristics of patients in GSE62254

Clinical characteristics	SCN7A		Chi-square	*p*-value
	Low	High		
Gender				
Female	38	63	9.329	0.002**
Male	112	87		
Age				
≤ 60	39	78	21.311	< 0.001***
> 60	111	72		
TNM-T				
T1–2	113	73	22.888	< 0.001***
T3–4	36	76		
Tx	1	1		
TNM-N				
N0	27	11	7.714	0.005**
N1–3	123	139		
TNM-M				
M0	140	133	1.994	0.158
M1	10	17		
Stage				
Stage I–II	79	47	14.080	< 0.001***
Stage III–IV	70	102		
NA	1	1		

**p < 0.01; ***p < 0.001

### Relationship between hub gene and tumor infiltrating immune cells

**Fig. 6:**
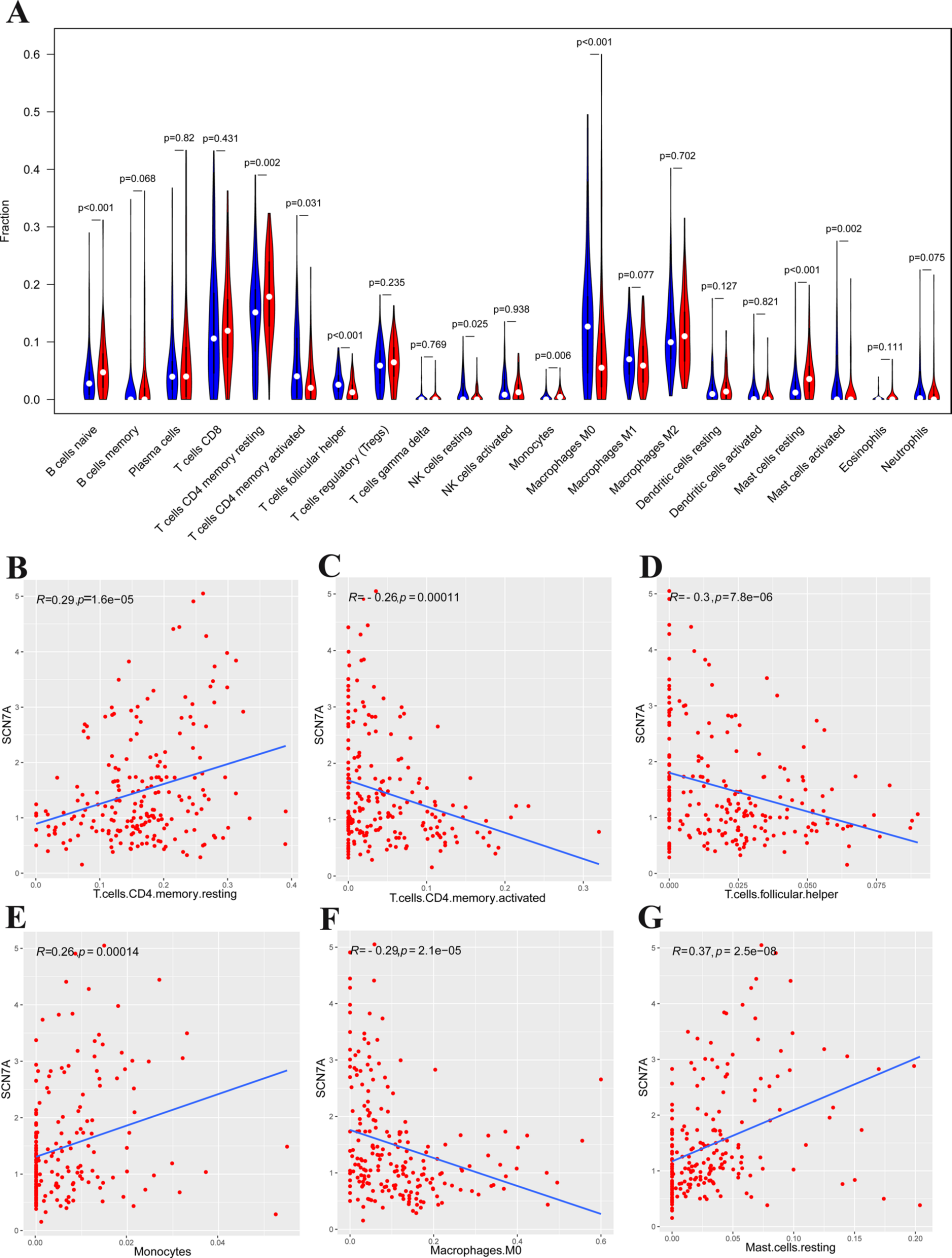
**A** The fractions of 21 immune cells between SCN7A low (blue) and high (red) samples. **B**–**G** The correlation between SCN7A expression levels and TIICs

Study suggested that TMB was related to the efficacy of immunotherapy in most cancer types. Hence, we calculated the fraction of tumor infiltrating immune cells in GC through CIBERSORT algorithm. The samples in TCGA were divided into two groups (high and low) by average expression of SCN7A. Figure 6A indicated that the fractions of several immune cells were different between SCN7A low (blue) and high (red) samples. B cells naïve, T cells CD4 memory resting, T cells CD4 memory activated, T cells follicular helper, Macrophages M0, Mast cells resting, and Mast cells activated differed significantly between SCN7A low and high groups. To further analyze the effects of SCN7A in tumor-infiltrating immune cells, we calculated the correlation between SCN7A expression levels and TIICs. The expression level of SCN7A was positively correlated with the fraction of T cells CD4 memory resting (Fig. 6B), monocytes (Fig. 6E), and mast cells resting (Fig. 6G) while negatively correlated with fractions of T cells CD4 memory activated (Fig. 6C), T cells follicular helper (Fig. 6D), and macrophages M0 (Fig. 6F).

## Discussion

Gastric cancer is a disease leading to numerous deaths worldwide. Finding a biomarker which is enable to predict immune response in gastric cancer has great guiding significance for the treatment of tumors, with the development of immunotherapy and wide application in numerous neoplastic diseases. Currently, the prevailing view is that TMB can reflect the therapeutic effect of chemotherapy drugs on cancer to some extent, especially immune checkpoint inhibitors. For example, in patients with non-small cell lung cancer with high TMB (≥ 10 mut/MB), anti-PD-1/PD-L1 immunotherapy results in excellent outcomes regardless of PD-L1 expression levels In this study, we found that TMB of gastric cancer was the fourth highest among all kinds of cancer by bioinformatic analysis based on TCGA database, implying that GC could be considered as an immune-responsive tumor. Then we used WGCNA to cluster 4881 genes and got a module highly correlated with TMB (r =  − 0.33, *p* = 7e−10). In this module, we were interested in the selected hub gene SCN7A. According to the results of KEGG pathway analysis, we inferred that SCN7A could affect the digestion and absorption function of gastric epithelial cells. Meanwhile, based on the existing clinical data of 477 GC patients from TCGA database and the microarray of GEO database, the mRNA level of SCN7A was negatively correlated with its clinical prognosis, and the later the clinical stage of the patients, the higher its expression was. Moreover, Tumor immune cell infiltration results showed that low-expression group of SCN7A had more infiltration in T cells CD4 memory resting and macrophages M0, while less infiltration in T cells CD4 memory activated and monocytes, indicating that such immune cells and pathways play a dominant role in the tumor microenvironment and promote immune responses.

Sodium Voltage-Gated Channel Alpha Subunit 7 (SCN7A) encodes a protein that is a specific sodium channel [23]. The SCN alpha family encodes proteins which are alpha subunits of sodium channel, called Na_v_1 [24]. Voltage-gated sodium channels are essential for initiating and propagating action potentials after depolarization of the cell surface membrane in most neurons [25]. The SCN7A protein is called Na_x_ for not being voltage gated. But SCN7A appears to be the most basal part of the SCN alpha family [26]. By locating on the plasma membrane, ion channels can sense and respond to changes in the extracellular environment, thereby playing a key role in cell signal transduction and cancer progression [27]. Studies have identified Na_x_ (SCN7A) as a specific sodium sensor mainly distributed in skin and other epithelial cells. Its upregulation mostly occurs in scars and skin inflammation and can activate the major sodium channel ENaC as well as the downstream inflammatory signal prostaglandin E2, while also regulating other pathways such as PAR-2 to promote the release of inflammatory factors IL-1 β and IL-8 [28]. Among them, IL-8 presents an inhibitory effect on adaptive immunity, affecting antigen presentation and the antitumor activity of effector T cells. The proportion of IL-8-producing myeloid cells and lymphocytes was higher in the resistant patients compared to those responding to immunotherapy, suggesting that high IL-8 is likely to result in suppression of antitumor immunity [29, 30]. In our study, we found that low expression of SCN7A was related to an increase in TMB. Tumors with high TMB imply the exposure of more neoantigens and thus are more prone to trigger T cell dependent immune responses to inhibit tumor development [31]. Therefore, we speculate that low expression of SCN7A improve the prognosis of patient survival by enhancing the response to immunotherapy.

Although much attention has been paid to the role of ion channels in excitable cells, regulation of cell membrane repolarization by ion flow and maintenance of resting potential are also present at the cell membrane of lymphocytes and other immune cells, and are important for maintaining normal electrophysiological function and immune activity [32]. We observed that SCN7A expression was associated with increased memory CD4 T cell and monocyte infiltration. Activation of CD4 + lymphocytes with a memory phenotype enables immune cells to mount a memory response to tumor antigens, thereby preventing tumor recurrence in patients with different cancers, which is predictive of longer survival [33]. As an important part of the innate immune system, mononuclear phagocyte system plays many functions such as immune defense, immune homeostasis, immune surveillance, antigen presentation, and immune regulation in body fluids and tissues [34]. But when tumorigenesis occurs in humans, as CD47 is expressed on the surface of cancer cells, macrophages not only fail to recognize and engulf it but promote it to complete metastasis in the super-early stage [35]. Studies in colon cancer mouse model have found that macrophages can phagocytose anti–PD-1 antibodies injected into mice, implying that macrophages influence the efficacy of immunochemotherapy drugs [36]. Therefore, we reasoned that expression level of SCN7A cause changes in gastric cancer infiltrating immune cells to promote antitumor immune efficacy.

In conclusion, our study demonstrated that SCN7A mutations in gastric cancer may be associated with higher TMB and improved patient outcomes. In addition, the altered expression of SCN7A affected the signaling pathways of the immune system and affected the antitumor immune response. These findings reveal the possibility that SCN7A is a novel biomarker and able to predict immune responses.

## Supplementary Information

**Additional file 1: Figure S2**. The process of soft threshold selection through network topology analysis.

**Additional file 2: Figure S1**. The level of TMB in differnt types of tumors.

## Data Availability

The data sets used and/or analysed during the current study are available from the corresponding author on reasonable request. The public data source: TCGA database (https://portal.gdc.cancer.gov/), GEO database (https://www.ncbi.nlm.nih.gov/geo/) and GEPIA2 database (http://gepia2.cancer-pku.cn).
