# Supplementary figures and images for: Identification of SCN7A as the key gene associated with tumor mutation burden in gastric cancer
Source: BMC Gastroenterol. 2022 Feb 5;22:45. doi: 10.1186/s12876-022-02112-4 (PMC8817579; doi:10.1186/s12876-022-02112-4)

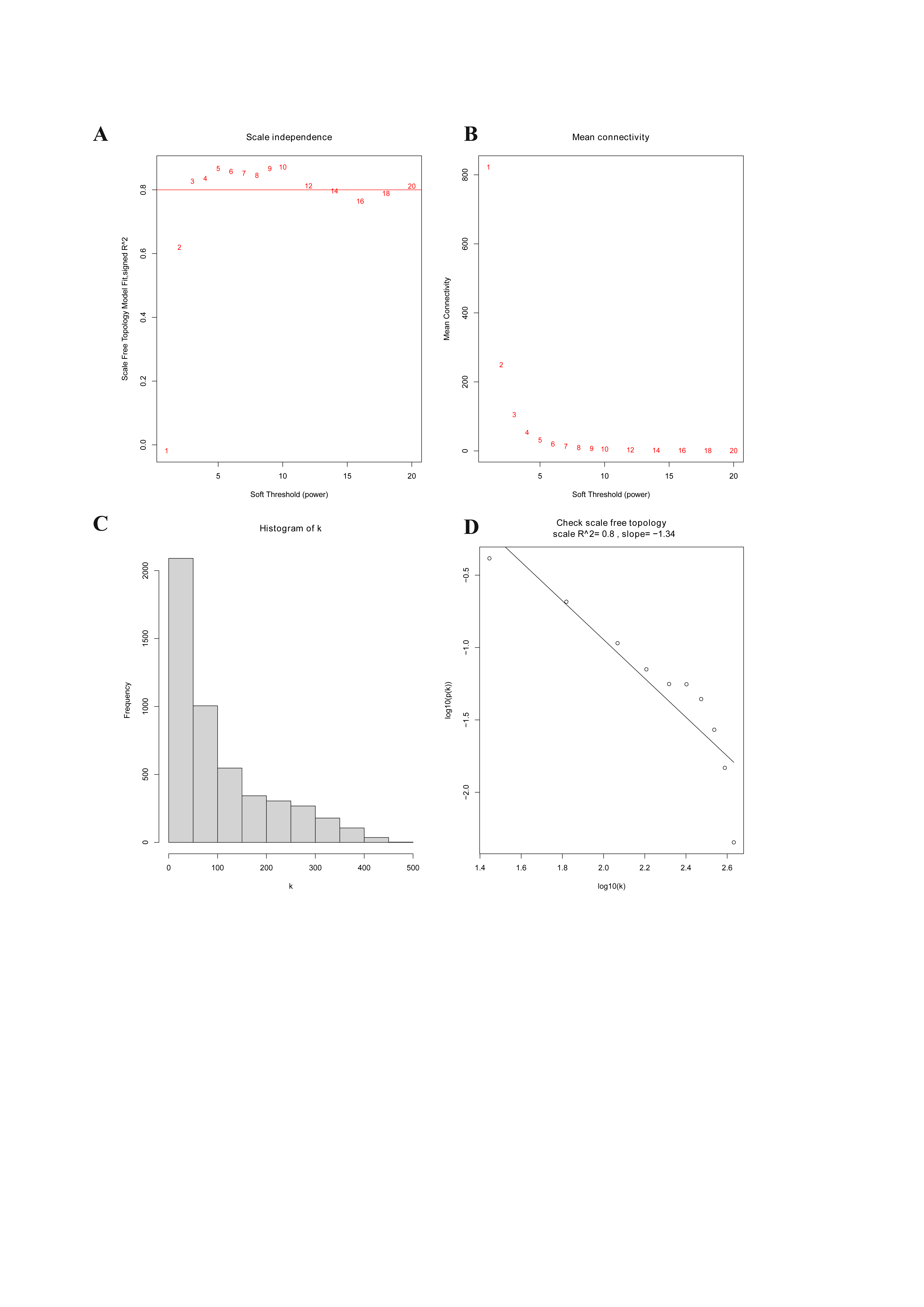

Supplement: Supplementary file 1 — Additional file 1: Figure S2. The process of soft threshold selection through network topology analysis. [file 12876_2022_2112_MOESM1_ESM.tif]

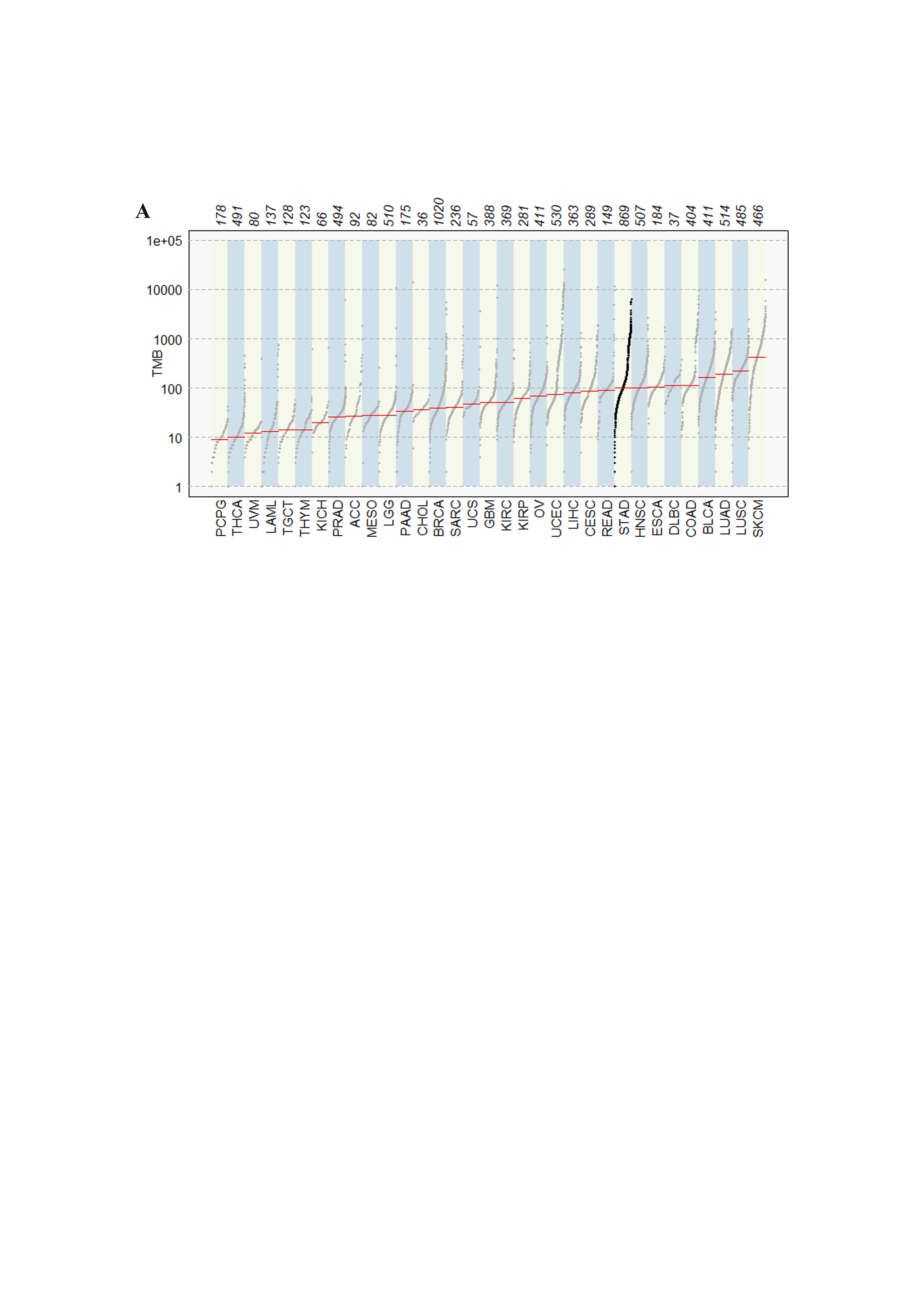

Supplement: Supplementary file 2 — Additional file 2: Figure S1. The level of TMB in differnt types of tumors. [file 12876_2022_2112_MOESM2_ESM.tif]
